# Bayesian Estimation of the DINA Model With Pólya-Gamma Gibbs Sampling

**DOI:** 10.3389/fpsyg.2020.00384

**Published:** 2020-03-10

**Authors:** Zhaoyuan Zhang, Jiwei Zhang, Jing Lu, Jian Tao

**Affiliations:** ^1^Key Laboratory of Applied Statistics of MOE, School of Mathematics and Statistics, Northeast Normal University, Changchun, China; ^2^Key Lab of Statistical Modeling and Data Analysis of Yunnan Province, School of Mathematics and Statistics, Yunnan University, Kunming, China

**Keywords:** Bayesian estimation, cognitive diagnosis models, DINA model, Pólya-Gamma Gibbs sampling algorithm, Metropolis-Hastings algorithm, potential scale reduction factor

## Abstract

With the increasing demanding for precision of test feedback, cognitive diagnosis models have attracted more and more attention to fine classify students whether has mastered some skills. The purpose of this paper is to propose a highly effective Pólya-Gamma Gibbs sampling algorithm (Polson et al., 2013) based on auxiliary variables to estimate the deterministic inputs, noisy “and” gate model (DINA) model that have been widely used in cognitive diagnosis study. The new algorithm avoids the Metropolis-Hastings algorithm boring adjustment the turning parameters to achieve an appropriate acceptance probability. Four simulation studies are conducted and a detailed analysis of fraction subtraction data is carried out to further illustrate the proposed methodology.

## 1. Introduction

Modeling the interaction between examinee's latent discrete skills (attributes) and items at the item level for binary response data, cognitive diagnosis models (CDMs) is an important methodology to evaluate whether the examinees have mastered multiple fine-grained skills, and these models have been widely used in a variety of the educational and psychological researches (Tatsuoka, 1984, 2002; Doignon and Falmagne, 1999; Maris, 1999; Junker and Sijtsma, 2001; de la Torre and Douglas, 2004; Templin and Henson, 2006; DiBello et al., 2007; Haberman and von Davier, 2007; de la Torre, 2009, 2011; Henson et al., 2009; von Davier, 2014; Chen et al., 2015). With the increasing complexity of the problems in cognitive psychology research, various specific and general formulations of CDMs have been proposed to deal with the practical problems. There are several specific CDMs, widely known among them, are the deterministic inputs, noisy “and” gate model (DINA; Junker and Sijtsma, 2001; de la Torre and Douglas, 2004; de la Torre, 2009), the noisy inputs, deterministic, “and” gate model (NIDA; Maris, 1999), the deterministic input, noisy “or” gate model (DINO; Templin and Henson, 2006) and the reduced reparameterized unified model (rRUM; Roussos et al., 2007). In parallel with the specific CDMs, the general CDMs have also made great progress, including the general diagnostic model (GDM; von Davier, 2005, 2008), the log-linear CDM (LCDM; Henson et al., 2009), and the generalized DINA (G-DINA; de la Torre, 2011). Parameter estimation has been a major concern in the application of CDMs. In fact, simultaneous estimations of items and examinee's latent discrete skills result in statistical complexities in the estimation task.

Within a fully Bayesian framework, a novel and highly effective Pólya-Gamma Gibbs sampling algorithm (PGGSA; Polson et al., 2013) based on the auxiliary variables is proposed to estimate the commonly used DINA model in this paper. The PGGSA overcomes the disadvantages of Metropolis-Hastings algorithm (Metropolis et al., 1953; Hastings, 1970; Chib and Greenberg, 1995; Chen et al., 2000), which requires to repeatedly adjust the specification of tuning parameters to achieve a certain acceptance probability and thus increases the computational burden. More specifically, the Metropolis–Hasting algorithm depends on the variance (tuning parameter) of the proposal distribution and is sensitive to step size. If the step size is too small, the chain will take longer to traverse the target density. If the step size is too large, there will be inefficiencies due to a high rejection rate. In addition, the Metropolis-Hastings algorithm is relatively difficulty to sample parameters with monotonicity or truncated interval restrictions. Instead, it can improve the accuracy of parameter estimation by employing strong informative prior distributions to avoid violating the restriction conditions (Culpepper, 2016).

The rest of this paper is organized as follows. Section 2 contains a short introductions of DINA model, its reparameterized form, and model identifications. A detailed implementation of PGGSA is shown in section 3. In section 4, four simulations focus on the performance of parameter recovery for the PGGSA, the results of comparing with the Metropolis-Hastings algorithm, the analysis of sensitivity of prior distributions for the PGGSA, the results of comparing with Culpepper (2015)'s Gibbs algorithm on the attribute classification accuracy and the estimation accuracy of class membership probability parameters. In addition, the quality of PGGSA is investigated using a fraction subtraction test data in section 5. We conclude the article with a brief discussion in section 6.

## 2. Models and Model Identifications

The DINA model focuses on whether the examinee *i* has mastered the *k* attribute, where *i* = 1, …, *N, k* = 1, …, *K*. Let α_*ik*_ be a dichotomous latent attribute variable with values of 0 or 1 indicating absence or presence of a attribute, respectively. $\alpha_{i}={\left(\alpha_{i1},\alpha_{i2},\ldots ,\alpha_{iK}\right)}^{\prime }$ is a vector of *K* dimensional latent attributes for the *i*th examinee. Given the categorical nature of the latent classes, ***α***_*i*_ belongs to one of *C* = 2^*K*^ attribute latent classes. If the *i*th examinee belongs to the *c*th classification, the attribute vector can be expressed as $\alpha_{c}={\left(\alpha_{c1},\alpha_{c2},\ldots ,\alpha_{cK}\right)}^{\prime }.$ Considering a test consisting of *J* items, each item *j* is associated with a vector of *K* dimensional item attributes, $q_{j}={\left(q_{j1},\ldots ,q_{jK}\right)}^{\prime }$, where

$$q_{jk}=\{\begin{array}{cc} \;1, & \text{if attribute }k\text{ is required by item }j,\\ \;0, & \text{if attribute }k\text{ is not required by item }j. \end{array}$$

Therefore, a ***Q*** matrix, ***Q* =**{*q*_*jk*_}_*J* × *K*_, can be obtained by the *J* item attribute vectors. The DINA model is conjunctive. That is, the examinee *i* must possess all the required attributes to answer the item *j* correctly. The ideal response pattern η_*ij*_ can be defined as follows

$$\eta_{ij}=\{\begin{array}{cc} \;1, & \text{if the examinee }i\text{ possesses all the required}\\ \; & \text{attributes for the item }j,\\ \;0, & \text{if the examinee }i\text{ does not master at least one}\\ \; & \text{attribute for the item }j. \end{array}$$

$\eta_{ij}=\text{ I }\left(\alpha_{i}^{\prime }q_{j}=q_{j}^{\prime }q_{j}\right)=\prod_{k=1}^{K}\alpha_{ik}^{q_{jk}}$, where I(·) denotes the indicator function. The parameters for a correct response to item *j* when given η_*ij*_ are denoted by *s*_*j*_ and *g*_*j*_. The slipping parameter *s*_*j*_ and the guessing parameter *g*_*j*_ refer to the probability of incorrectly answering the item when η_*ij*_ = 1 and the probability of correctly guessing the answer when η_*ij*_ = 0, respectively. Let *Y*_*ij*_ denote the observed item response for the *i*th examinee to response *j*th item, *Y*_*ij*_ = 1 if the *i*th examinee correct answer the *j*th item, 0 otherwise. The parameters *s*_*j*_ and *g*_*j*_ are formally defined by

$$s_{j}=p\left(Y_{ij}=0|\eta_{ij}=1\right)\text{ and }g_{j}=p\left(Y_{ij}=1|\eta_{ij}=0\right).$$

The probabilities of observing response given attributes ***α*** are represented by

(1) $$\begin{array}{l} \;f_{ij}=p\left(Y_{ij}=1|\alpha_{i},s_{j},g_{j}\right)={\left(1-s_{j}\right)}^{\eta_{ij}}\\ g_{j}^{1-\eta_{ij}}=\left\{ \begin{array}{cc} \;1-s_{j}, & \eta_{ij}=\overset{K}{\underset{k=1}{\prod }}\alpha_{ik}^{q_{jk}}=1,\\ \;g_{j}, & \eta_{ij}=\overset{K}{\underset{k=1}{\prod }}\alpha_{ik}^{q_{jk}}=0. \end{array}\right. \end{array}$$

and.

(2) $$\begin{array}{l} h_{ij}=1-p\left(Y_{ij}=1|\alpha_{i},s_{j},g_{j}\right)=\left[1-{\left(1-s_{j}\right)}^{\eta_{ij}}g_{j}^{1-\eta_{ij}}\right]\\ \;=\left\{ \begin{array}{cc} \;s_{j}, & \eta_{ij}=\overset{K}{\underset{k=1}{\prod }}\alpha_{ik}^{q_{jk}}=1,\\ \;1-g_{j}, & \eta_{ij}=\overset{K}{\underset{k=1}{\prod }}\alpha_{ik}^{q_{jk}}=0. \end{array}\right. \end{array}$$

### 2.1. The Reparameterized DINA Model

To describe the relationship between the attribute vector and the observed response, we can reexpress the DINA model as follow:

(3) $$\begin{array}{l} p\left(Y_{ij}=1|\alpha_{i}\right)=g_{j}+\left(1-s_{j}-g_{j}\right)\overset{K}{\underset{k=1}{\prod }}\alpha_{ik}^{q_{jk}}, \end{array}$$

where the model discrimination index can be defined as 1 − *s*_*j*_ − *g*_*j*_ = IDI_*j*_ (de la Torre, 2008). Based on the traditional DINA model, we reparameterize *s*_*j*_ and *g*_*j*_ from the probability scale to the logit scale (Henson et al., 2009; DeCarlo, 2011; von Davier, 2014; Zhan et al., 2017). That is,

$$\zeta_{j}=\text{logit}\left(g_{j}\right),$$

$$\beta_{j}=\text{logit}\left(1-s_{j}\right)-\text{logit}\left(g_{j}\right),$$

where logit(*x*) = log(*x*/(1 − *x*)). Therefore, the reparameterized DINA model (DeCarlo, 2011) can be written as

(4) $$\begin{array}{l} \text{logit}\left[p\left(Y_{ij}=1|\alpha_{i},\varsigma_{j},\beta_{j}\right)\right]=\varsigma_{j}+\beta_{j}\overset{K}{\underset{k=1}{\prod }}\alpha_{ik}^{q_{jk}}, \end{array}$$

where ς_*j*_ and β_*j*_ are the item intercept and interaction parameters, respectively.

### 2.2. The Likelihood Function of the Reparameterized DINA Model Based on the Latent Class

Suppose that the vector of item responses for the *i*th examinee can be denoted as $Y_{i}={\left(Y_{i1},\ldots ,Y_{iJ}\right)}^{\prime }.$ Let the vector of intercept and interaction parameters for *J* items be ***ς*** and ***β***, where ***ς =*** (ς_1_, …, ς_*J*_) and ***β =*** (β_1_, …, β_*J*_). Given the categorical nature of the latent classes, ***α***_*i*_ belongs to one of *C* = 2^*K*^ attribute latent classes. For the *i*th examinee belonging to the *c*th classification, the attribute vector is expressed as $\alpha_{c}={\left(\alpha_{c1},\alpha_{c2},\ldots ,\alpha_{cK}\right)}^{\prime }.$ According the Equation (4), the probability of observing ***Y***_*i*_ that the *i*th examinee belonging to the *c*th latent class answers *J* items can be written as

(5) $$\begin{array}{l} p\left(Y_{i}|\alpha_{i}=\alpha_{c},\varsigma ,\beta \right)=\overset{J}{\underset{j=1}{\prod }}\\ {\left[p\left(Y_{ij}=1|\alpha_{c},\varsigma_{j},\beta_{j}\right)\right]}^{Y_{ij}}{\left[1-p\left(Y_{ij}=1|\alpha_{c},\varsigma_{j},\beta_{j}\right)\right]}^{1-Y_{ij}}. \end{array}$$

where ***α***_*i*_ = ***α***_*c*_ denotes the examinee *i* belongs to the *c*th latent class. *p*(*Y*_*ij*_ = 1|***α***_*c*_, ς_*j*_, β_*j*_) is the probability that the examinee *i* in class *c* correctly answers the item *j*.

Let π_*c*_ = *p*(***α***_*c*_) be the probability of examinees for each class *c*, *c* = 1, …, *C*, and ***π*** $={\left(\pi_{1},\ldots ,\pi_{C}\right)}^{\prime }$ is *C* dimensional vector of class membership probabilities, where $\overset{C}{\underset{c=1}{\sum }}\pi_{c}=1$. Therefore, the probability of observing ***Y***_*i*_ given item parameters ***ς***, ***β*** and class membership probabilities ***π*** can be written as

(6) $$\begin{array}{l} p\left(Y_{i}|\varsigma ,\beta ,\pi \right)=\overset{C}{\underset{c=1}{\sum }}\pi_{c}p\left(Y_{i}|\alpha_{i}=\alpha_{c},\varsigma ,\beta \right). \end{array}$$

The likelihood function based on the latent class can be written as

(7) $$\begin{array}{l} p\left(Y|\varsigma ,\beta ,\pi \right)=\overset{N}{\underset{i=1}{\prod }}\overset{C}{\underset{c=1}{\sum }}\pi_{c}p\left(Y_{i}|\alpha_{i}=\alpha_{c},\varsigma ,\beta \right). \end{array}$$

### 2.3. Model Identification

The model identification is an important cornerstone for estimating parameters and practical applications. Chen et al. (2015); Xu and Zhang (2016), and Xu (2017) discuss the DINA model identification conditions. Gu and Xu (2019) further provide a set of sufficient and necessary conditions for the identifiability of the DINA model. That is,

**Condition 1:** *(1) The* *Q-matrix is complete under the DINA model and without loss of generality, we assume the* *Q-matrix takes the following form:*

(8) $$\begin{array}{l} Q={\left(\begin{array}{c} I_{K}\\ Q^{*} \end{array}\right)}_{J\times K}, \end{array}$$

*where* $I_{K}$ *is the* *K* × *K* *identify matrix and* Q* *is a* (*J* − *K*) × *K* *submarix of* *Q*.

*(2) Each of the* *K* *attributes is required by at least three items*.

**Condition 2**: *Any two different columns of the submatrix* Q* *in (8) are distinct*.

Under the above two conditions, Gu and Xu (2019) give the following identifiability result.

**Theorem** (Sufficient and Necessary Condition) *Conditions* 1 *and* 2 *are sufficient and necessary for the identifiability of all the DINA model parameters*.

## 3. Pólya-Gamma Gibbs Sampling Algorithm

Polson et al. (2013) propose a new data augmentation strategy for fully Bayesian inference in logistic regression. The data augmentation approach appeals to a new class of Pólya-Gamma distribution rather than Albert and Chib (1993)'s data augmentation algorithm based on a truncated normal distribution. Next, we introduce the Pólya-Gamma distribution.

**Definition**: Let ${\left\{T_{k}\right\}}_{k=1}^{+\infty }$ is a iid random variable sequences from a Gamma distribution with parameters λ and 1. That is, *T*_*k*_ ~ Gamma (λ, 1). A random variable *W* follows a Pólya-Gamma distribution with parameters λ > 0 and τ ∈ *R*, denoted *W* ~ PG(λ, τ), if

(9) $$\begin{array}{l} W\overset{D}{=}\frac{1}{2\pi }\overset{+\infty }{\underset{k=1}{\sum }}\frac{T_{k}}{{\left(k-\frac{1}{2}\right)}^{2}+\frac{\tau^{2}}{4\pi^{2}}}, \end{array}$$

where $\overset{D}{=}$ denotes equality in distribution. In fact, the Pólya-Gamma distribution is an infinite mixture of gamma distributions which provide the plausibility to sample from Gamma distributions.

Based on Polson et al. (2013, p. 1341, Equation 7)'s Theorem 1, the likelihood contribution of the *i*th examinee to answer the *j*th item can be expressed as

(10) $$\begin{array}{l} L\left(\varsigma_{j},\beta_{j},\alpha_{i}\right)=\frac{{\left[\exp\left(\varsigma_{j}+\beta_{j}\overset{K}{\underset{k=1}{\prod }}\alpha_{ik}^{q_{jk}}\right)\right]}^{Y_{ij}}}{1+\exp\left(\varsigma_{j}+\beta_{j}\overset{K}{\underset{k=1}{\prod }}\alpha_{ik}^{q_{jk}}\right)}\\ \;\propto \exp\left[k_{ij}\left(\varsigma_{j}+\beta_{j}\overset{K}{\underset{k=1}{\prod }}\alpha_{ik}^{q_{jk}}\right)\right]\\ \;\times \;\overset{\infty }{\underset{0}{\int }}\exp\left[-\frac{W_{ij}{\left(\varsigma_{j}+\beta_{j}\overset{K}{\underset{k=1}{\prod }}\alpha_{ik}^{q_{jk}}\right)}^{2}}{2}\right]\\ \;p\left(W_{ij}|1,0\right)dW_{ij}, \end{array}$$

where $k_{ij}=Y_{ij}-\frac{1}{2}.$ *p*(*W*_*ij*_ | 1, 0) is the conditional density of *W*_*ij*_. That is, *W*_*ij*_ ~ PG(1, 0). The auxiliary variable *W*_*ij*_ follows a Pólya-Gamma distribution with parameters (1, 0). Biane et al. (2001) provide proofs of Equation (10). In addition, Polson et al. (2013) further discuss Equation (10). Therefore, the full conditional distribution of ***ς***, ***β***, ***α*** given the auxiliary variables *W*_*ij*_ can be written as

(11) $$\begin{array}{l} \;p\left(\varsigma ,\beta_{j},\alpha |W,Y\right)\propto \\ \;\{\overset{N}{\underset{i=1}{\prod }}\overset{J}{\underset{j=1}{\prod }}[\exp\left[k_{ij}\left(\varsigma_{j}+\beta_{j}\overset{K}{\underset{k=1}{\prod }}\alpha_{ik}^{q_{jk}}\right)\right]\\ \;\exp\left[-\frac{W_{ij}{\left(\varsigma_{j}+\beta_{j}\overset{K}{\underset{k=1}{\prod }}\alpha_{ik}^{q_{jk}}\right)}^{2}}{2}\right]]\}\\ \times \left\{\overset{J}{\underset{j=1}{\prod }}\left[p\left(\varsigma_{j}\right)p\left(\beta_{j}\right)\right]\right\}\left\{\overset{N}{\underset{i=1}{\prod }}p\left(\alpha_{i}\right)\right\}. \end{array}$$

where *p*(ς), *p*(β), and *p*(***α***) are the prior distributions, respectively. The joint posterior distribution based on the latent classes is given by

$$\begin{array}{l} p\left(\varsigma ,\beta_{j},\alpha ,\pi ,W|Y\right)\propto \{\overset{N}{\underset{i=1}{\prod }}\overset{J}{\underset{j=1}{\prod }}\overset{C}{\underset{c=1}{\prod }}[p\left(Y_{ij}=y_{ij}|\varsigma_{j},\beta_{j},\alpha_{i}=\alpha_{c}\right)\\ \;f\left(W_{ij}|\varsigma_{j},\beta_{j},\alpha_{i}=\alpha_{c}\right)]\}\\ \;\times \left\{\overset{J}{\underset{j=1}{\prod }}\left[p\left(\varsigma_{j}\right)p\left(\beta_{j}\right)\right]\right\}\left\{\overset{C}{\underset{c=1}{\prod }}p\left(\pi_{c}\right)\right\}. \end{array}$$

where *p*(ς), *p*(β), and *p*(π) are the prior distributions, respectively.

**Step 1**: Sampling the auxiliary variable *W*_*ij*_, given the item intercept and interaction parameters ς_*j*_, β_*j*_ and ***α***_*i*_ = ***α***_*c*_. According to Equation (10), the full conditional posterior distribution of the random auxiliary variable *W*_*ij*_ is given by

(12) $$\begin{array}{l} f\left(W_{ij}|\varsigma_{j},\beta_{j},\alpha_{i}=\alpha_{c}\right)\propto \exp\left[-\frac{W_{ij}{\left(\varsigma_{j}+\beta_{j}\overset{K}{\underset{k=1}{\prod }}\alpha_{ik}^{q_{jk}}\right)}^{2}}{2}\right]\\ \;p\left(W_{ij}|1,0\right), \end{array}$$

According to Biane et al. (2001) and Polson et al. (2013; p. 1341), the density function *p*(*W*_*ij*_|1, 0) can be written as

(13) $$\begin{array}{l} p\left(W_{ij}|1,0\right)=\overset{\infty }{\underset{v=0}{\sum }}{\left(-1\right)}^{v}\frac{\left(2k+1\right)}{\sqrt{2\pi W_{ij}}}\exp\left[-\frac{{\left(2k+1\right)}^{2}}{8W_{ij}}\right]. \end{array}$$

Therefore, *f*(*W*_*ij*_ | ς_*j*_, β_*j*_, ***α***_*i*_ = ***α***_*c*_) is proportional to

(14) $$\begin{array}{l} \overset{\infty }{\underset{v=0}{\sum }}{\left(-1\right)}^{v}\frac{\left(2k+1\right)}{\sqrt{2\pi W_{ij}}}\exp\left[-\frac{{\left(2k+1\right)}^{2}}{8W_{ij}}-\frac{W_{ij}{\left(\varsigma_{j}+\beta_{j}\overset{K}{\underset{k=1}{\prod }}\alpha_{ik}^{q_{jk}}\right)}^{2}}{2}\right]. \end{array}$$

Finally, the specific form of the full conditional distribution of *W*_*ij*_ is as follows

(15) $$\begin{array}{l} W_{ij}\sim \text{PG}\left(1,|\varsigma_{j}+\beta_{j}\overset{K}{\underset{k=1}{\prod }}\alpha_{ik}^{q_{jk}}|\right). \end{array}$$

Next, the Gibbs samplers are used to draw the item parameters.

**Step 2**: Sampling the intercept parameter ς_*j*_ for each item *j*. The prior distribution of ς_*j*_ is assumed to follow a normal distribution, that is, $\varsigma_{j}\sim N\left(\mu_{\varsigma },\sigma_{\varsigma }^{2}\right)$. Given ***Y***, ***W***, ***β***, and ***α***, the fully condition posterior distribution of ς_*j*_ is given by

(16) $$\begin{array}{l} p\left(\varsigma_{j}|Y,W,\alpha ,\beta_{j}\right)\propto \overset{N}{\underset{i=1}{\prod }}\{\frac{{\left[\exp\left(\varsigma_{j}+\beta_{j}\overset{K}{\underset{k=1}{\prod }}\alpha_{ik}^{q_{jk}}\right)\right]}^{Y_{ij}}}{1+\exp\left(\varsigma_{j}+\beta_{j}\overset{K}{\underset{k=1}{\prod }}\alpha_{ik}^{q_{jk}}\right)}\\ \;f\left(W_{ij}|\varsigma_{j},\beta_{j},\alpha_{i}=\alpha_{c}\right)\}p\left(\varsigma_{j}\right), \end{array}$$

where *f*(*W*_*ij*_ | ς_*j*_, β_*j*_, ***α***_*i*_ = ***α***_*c*_) is equal to the following equation (the details see Polson et al., 2013; p. 1341)

(17) $$\begin{array}{l} f\left(W_{ij}|\varsigma_{j},\beta_{j},\alpha_{i}=\alpha_{c}\right)=\left\{\cosh\left(2^{-1}|\varsigma_{j}+\beta_{j}\overset{K}{\underset{k=1}{\prod }}\alpha_{ik}^{q_{jk}}|\right)\right\}\frac{2^{0}}{\Gamma \left(1\right)}\\ \;\times \overset{\infty }{\underset{v=0}{\sum }}{\left(-1\right)}^{v}\frac{\left(2k+1\right)}{\sqrt{2\pi W_{ij}}}\exp\left[-\frac{{\left(2k+1\right)}^{2}}{8W_{ij}}-\frac{W_{ij}{\left(\varsigma_{j}+\beta_{j}\overset{K}{\underset{k=1}{\prod }}\alpha_{ik}^{q_{jk}}\right)}^{2}}{2}\right]. \end{array}$$

After rearrangement, the full conditional posterior distribution of ς_*j*_ can be written as follows

(18) $$\begin{array}{l} p\left(\varsigma_{j}|Y,W,\alpha ,\beta_{j}\right)\propto \overset{N}{\underset{i=1}{\prod }}\{\frac{{\left[\exp\left(\varsigma_{j}+\beta_{j}\overset{K}{\underset{k=1}{\prod }}\alpha_{ik}^{q_{jk}}\right)\right]}^{Y_{ij}}}{1+\exp\left(\varsigma_{j}+\beta_{j}\overset{K}{\underset{k=1}{\prod }}\alpha_{ik}^{q_{jk}}\right)}\\ \;\left[\cosh\left(2^{-1}|\varsigma_{j}+\beta_{j}\overset{K}{\underset{k=1}{\prod }}\alpha_{ik}^{q_{jk}}|\right)\right]\\ \;\times \exp\left[-\frac{{\left(\varsigma_{j}+\beta_{j}\overset{K}{\underset{k=1}{\prod }}\alpha_{ik}^{q_{jk}}\right)}^{2}W_{ij}}{2}\right]\}p\left(\varsigma_{j}\right). \end{array}$$

$$\text{Va}{\text{r}}_{\beta_{j}}\times \left(\mu_{\beta }\sigma_{\beta }^{-2}+\overset{N}{\underset{i=1}{\sum }}\left[{\left(\overset{K}{\underset{k=1}{\prod }}\alpha_{ik}^{q_{jk}}\right)}^{2}W_{ij}\right]\left(\frac{\overset{N}{\underset{i=1}{\sum }}\left(2Y_{ij}\overset{K}{\underset{k=1}{\prod }}\alpha_{ik}^{q_{jk}}-\overset{K}{\underset{k=1}{\prod }}\alpha_{ik}^{q_{jk}}-2\varsigma_{j}W_{ij}\overset{K}{\underset{k=1}{\prod }}\alpha_{ik}^{q_{jk}}\right)}{2\overset{N}{\underset{i=1}{\sum }}\left[{\left(\overset{K}{\underset{k=1}{\prod }}\alpha_{ik}^{q_{jk}}\right)}^{2}W_{ij}\right]}\right)\right)$$

Therefore, the fully condition posterior distribution of ς_*j*_ follow normal distribution with mean

$$\text{Va}{\text{r}}_{\varsigma_{j}}\times \left(\mu_{\varsigma }\sigma_{\varsigma }^{-2}+\left(\overset{N}{\underset{i=1}{\sum }}W_{ij}\right)\left(\frac{\overset{N}{\underset{i=1}{\sum }}2Y_{ij}-1-2\beta_{j}W_{ij}\overset{K}{\underset{k=1}{\prod }}\alpha_{ik}^{q_{jk}}}{2\overset{N}{\underset{i=1}{\sum }}W_{ij}}\right)\right),$$

and variance

$$\text{Va}{\text{r}}_{\varsigma_{j}}={\left(\sigma_{\varsigma }^{-2}+\left(\overset{N}{\underset{i=1}{\sum }}W_{ij}\right)\right)}^{-1}.$$

**Step 3**: Sampling the interaction parameter β_*j*_ for each item *j*. The prior distribution of β_*j*_ is assumed to follow a truncated normal distribution to satisfy the model identification restriction (Junker and Sijtsma, 2001; Henson et al., 2009; DeCarlo, 2012; Culpepper, 2015). That is, $\beta_{j}\sim N\left(\mu_{\beta },\sigma_{\beta }^{2}\right)\text{ I }\left(\beta_{j}>0\right).$ Similarly, given ***Y***, ***W***, ***ς***, and ***α***, the full condition posterior distribution of β_*j*_ is given by

(19) $$\begin{array}{l} p\left(\beta_{j}|Y,W,\alpha ,\varsigma \right)\propto \overset{N}{\underset{i=1}{\prod }}\{\frac{{\left[\exp\left(\varsigma_{j}+\beta_{j}\overset{K}{\underset{k=1}{\prod }}\alpha_{ik}^{q_{jk}}\right)\right]}^{Y_{ij}}}{1+\exp\left(\varsigma_{j}+\beta_{j}\overset{K}{\underset{k=1}{\prod }}\alpha_{ik}^{q_{jk}}\right)}\\ \;\left[\cosh\left(2^{-1}|\varsigma_{j}+\beta_{j}\overset{K}{\underset{k=1}{\prod }}\alpha_{ik}^{q_{jk}}|\right)\right]\\ \;\times \exp\left[-\frac{{\left(\varsigma_{j}+\beta_{j}\overset{K}{\underset{k=1}{\prod }}\alpha_{ik}^{q_{jk}}\right)}^{2}W_{ij}}{2}\right]\}p\left(\beta_{j}\right). \end{array}$$

Therefore, the fully condition posterior distribution of ς_*j*_ follow the truncated normal distribution with mean

and variance

(20) $$\begin{array}{l} \text{Va}{\text{r}}_{\beta_{j}}={\left\{\sigma_{\beta }^{-2}+\overset{N}{\underset{i=1}{\sum }}\left[{\left(\overset{K}{\underset{k=1}{\prod }}\alpha_{ik}^{q_{jk}}\right)}^{2}W_{ij}\right]\right\}}^{-1} \end{array}$$

**Step 4**: Sampling the attribute vector ***α***_*i*_ for each examinee *i*. Given ***Y***, ***W***, ***ς***, and ***β***, we can update the *i*th examinee's attribute vector ***α***_*i*_ from the following multinomial distribution

(21) $$\begin{array}{l} \alpha_{i}|Y_{i},W_{i},\varsigma ,\;\beta \sim \text{Multinomial }\left(1,\left[\lambda_{i1},\ldots ,\lambda_{iC}\right]\right). \end{array}$$

where the probability that the attribute vector ***α***_*i*_ belongs to the *c*th(*c* = 1, …, *C*) class can be written as

(22) $$\begin{array}{l} \lambda_{ic}=P\left(\alpha_{i}=\alpha_{c}|Y_{i},W_{i},\varsigma ,\beta ,\pi \right)\\ =\frac{\pi_{c}p\left(Y_{i}|\alpha_{i}=\alpha_{c},\varsigma ,\beta \right)f\left(W_{i}|\alpha_{i}=\alpha_{c},\varsigma ,\beta \right)}{\overset{C}{\underset{c=1}{\sum }}\pi_{c}p\left(Y_{i}|\alpha_{i}=\alpha_{c},\varsigma ,\beta \right)f\left(W_{i}|\alpha_{i}=\alpha_{c},\varsigma ,\beta \right)}. \end{array}$$

**Step 5**: Sampling the class membership probabilities ***π***. The prior of ***π*** is assumed to follow a Dirichlet distribution. I.e., ***π =*** (π_1_, …, π_*C*_) ~Dirichlet(δ_0_, …, δ_0_). The full condition posterior distribution of the class membership probabilities ***π*** can be written as

(23) $$\begin{array}{l} \pi |\alpha_{1},\ldots ,\alpha_{C}\sim \text{Dirichlet }(\delta_{0}+\overset{N}{\underset{i=1}{\sum }}\text{ I }\left(\alpha_{i}=\alpha_{1}\right),\ldots ,\delta_{0}\\ \;+\overset{N}{\underset{i=1}{\sum }}\text{ I }\left(\alpha_{i}=\alpha_{C}\right)). \end{array}$$

## 4. Simulation Study

### 4.1. Simulation 1

#### 4.1.1. Simulation Design

In this simulation study, the purpose is to assess the performance of the Pólya-Gamma Gibbs sampling algorithm. Considering the test length is *J* = 30, and the number of the attribute is set equal to *K* = 5. The *Q-*matrix is shown in Table 1, where the design of *Q-*matrix satisfies Gu and Xu (2019)'s DINA model identification conditions. For the true values of the class membership probabilities, we only consider the most general case that the class membership probabilities are flat though all class, i.e., $\pi_{c}=\frac{1}{2^{K}},$ *c* = 1, …, *C*, where *C* = 2^*K*^. Next, two factors and their varied test conditions are simulated. (a) two sample sizes (*N* = 1000, 2000) are considered; (b) Following Huebner and Wang (2011) and Culpepper (2015), four noise levels are considered to explore the relationship between noise level and recovery by constraining the true values of the item parameters. For each item, (b1) low noise level (LNL) case: *s*_*j*_ = *g*_*j*_ = 0.1; the corresponding true values of reparameterized parameters are ζ_*j*_ = −2.1972, β_*j*_ = 4.3945; (b2) high noise level (HNL) case : *s*_*j*_ = *g*_*j*_ = 0.2; the corresponding true values of reparameterized parameters are ζ_*j*_ = −1.3863, β_*j*_ = 2.7726; (b3) slipping higher than guessing (SHG) case: *s*_*j*_ = 0.2, *g*_*j*_ = 0.1; the corresponding true values of reparameterized parameters are ζ_*j*_ = −2.1972, β_*j*_ = 3.5835; (b4) guessing higher than slipping (GHS) case: *s*_*j*_ = 0.1, *g*_*j*_ = 0.2; the corresponding true values of reparameterized parameters are ζ_*j*_ = −1.3863, β_*j*_ = 3.5835. Fully crossing the different levels of these two factors yield 8 conditions.

**Table 1:** The Q matrix design in the simulation study 1.

	**Attribute Q(matrix)**						**Attribute Q(matrix)**				
**Item**	**α_1_**	**α_2_**	**α_3_**	**α_4_**	**α_5_**	**Item**	**α_1_**	**α_2_**	**α_3_**	**α_4_**	**α_5_**
1	1	0	0	0	0	16	0	1	0	1	0
2	0	1	0	0	0	17	0	1	0	0	1
3	0	0	1	0	0	18	0	0	1	1	0
4	0	0	0	1	0	19	0	0	1	0	1
5	0	0	0	0	1	20	0	0	0	1	1
6	1	0	0	0	0	21	1	1	1	0	0
7	0	1	0	0	0	22	1	1	0	1	0
8	0	0	1	0	0	23	1	1	0	0	1
9	0	0	0	1	0	24	1	0	1	1	0
10	0	0	0	0	1	25	1	0	1	0	1
11	1	1	0	0	0	26	1	0	0	1	1
12	1	0	1	0	0	27	0	1	1	1	0
13	1	0	0	1	0	28	0	1	1	0	1
14	1	0	0	0	1	29	0	1	0	1	1
15	0	1	1	0	0	30	0	0	0	1	1

#### 4.1.2. Priors

Based on the four noise levels, the corresponding four kinds of non-informative prior are used. I.e.,

$$\begin{array}{l} \left(b1\right)\;\zeta_{j}\sim N\left(-2.1972,10^{5}\right),\beta_{j}\sim N\left(4.3945,10^{5}\right)\text{ I }\left(\beta_{j}>0\right);\\ \left(b2\right)\;\zeta_{j}\sim N\left(-1.3863,10^{5}\right),\beta_{j}\sim N\left(2.7726,10^{5}\right)\text{ I }\left(\beta_{j}>0\right);\\ \left(b3\right)\;\zeta_{j}\sim N\left(-2.1972,10^{5}\right),\beta_{j}\sim N\left(3.5835,10^{5}\right)\text{ I }\left(\beta_{j}>0\right);\\ \left(b4\right)\;\zeta_{j}\sim N\left(-1.3863,10^{5}\right),\beta_{j}\sim N\left(3.5835,10^{5}\right)\text{ I }\left(\beta_{j}>0\right), \end{array}$$

where the purpose of using non-informative priors is to eliminate the influence of prior uncertainty on posterior inferences. Similarly, the non-informative Dirichlet prior distribution is employed for the class membership probabilities ***π***. I.e., (π_1_, …, π_*C*_) ~ Dirichlet (1, …, 1).

#### 4.1.3. Convergence Diagnostics

As an illustration of the convergence of parameter estimates, we only consider the low noise level (LNL) case and the number of examinees is 1,000. Two methods are used to check the convergence of parameter estimates. One is the “eyeball” method to monitor the convergence by visually inspecting the history plots of the generated sequences (Hung and Wang, 2012; Zhan et al., 2017), and another method is to use the Gelman-Rubin method (Gelman and Rubin, 1992; Brooks and Gelman, 1998) to check the convergence of parameter estimates.

**Figure 1:**
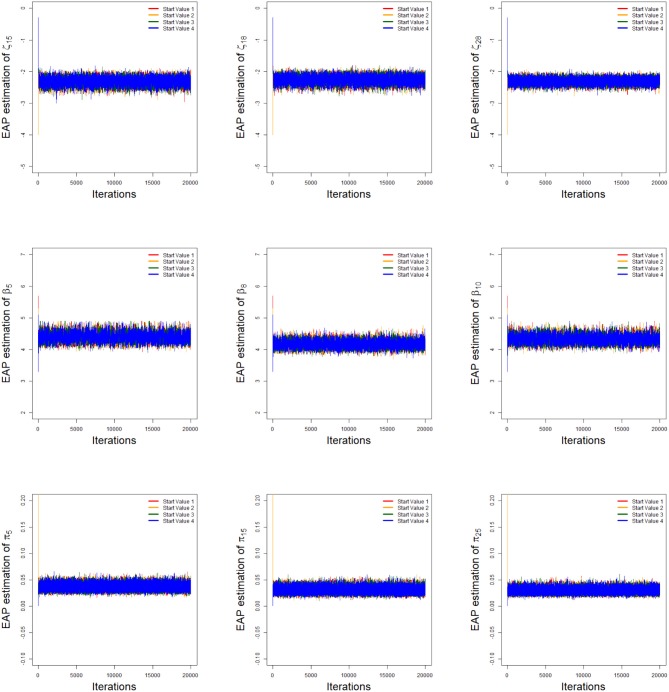
The trace plots of the arbitrarily selected item and class membership probability parameters.

To implement the MCMC sampling algorithm, chains of length 20,000 with an initial burn-in period 10,000 are chosen. Four chains started at overdispersed starting values are run for each replication. The trace plots of Markov Chains for three randomly selected items and class membership probabilities are shown in Figure 1. In addition, the potential scale reduction factor (PSRF; Brooks and Gelman, 1998) values of all parameters are <1.1, which ensures that all chains converge as expected. The trace plots of PSRF values are shown in the simulation 2.

#### 4.1.4. Evaluation Criteria for Convergence and Accuracy of Parameter Estimations

The accuracy of the parameter estimates is measured by two evaluation criteria, i.e., Bias and Mean Squared Error (MSE). Let η be the interested parameter. Assume that *M* = 25 data sets are generated. Also, let ${\hat{\eta }}^{\left(m\right)}$ be the posterior mean obtained from the *m*th simulated data set for *m* = 1, …, *M*.

The Bias for parameter is defined as

(24) $$\begin{array}{l} \text{Bias }\left(\eta \right)=\frac{1}{M}\overset{M}{\underset{m=1}{\sum }}\left({\hat{\eta }}^{\left(m\right)}-\eta \right), \end{array}$$

and the MSE for parameter is defined as

(25) $$\begin{array}{l} \text{MSE }\left(\eta \right)=\frac{1}{M}\overset{M}{\underset{m=1}{\sum }}{\left({\hat{\eta }}^{\left(m\right)}-\eta \right)}^{2}. \end{array}$$

**Figure 2:**
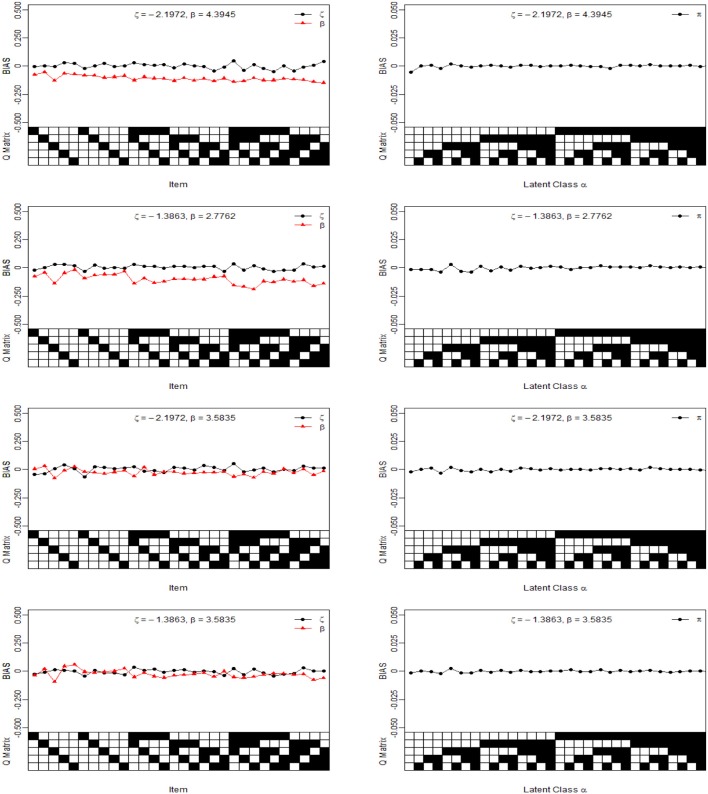
The Bias of intercept, interaction and the latent class parameters under four different noise levels. The *Q* Matrix denotes the skills required for each item along the *x* axis, where the black square = “1” and white square = “0.” The α_*ck*_ denotes the examinee who belongs to the *c*th latent class whether has mastered *k*th skill, where the black square = “1” for the presence of a skill and white square = “0” for the absence of a skill, $\alpha_{c}={\left(\alpha_{c1},\ldots ,\alpha_{cK}\right)}^{\prime }$. Note the Bias values are estimated from 25 replications.

**Figure 3:**
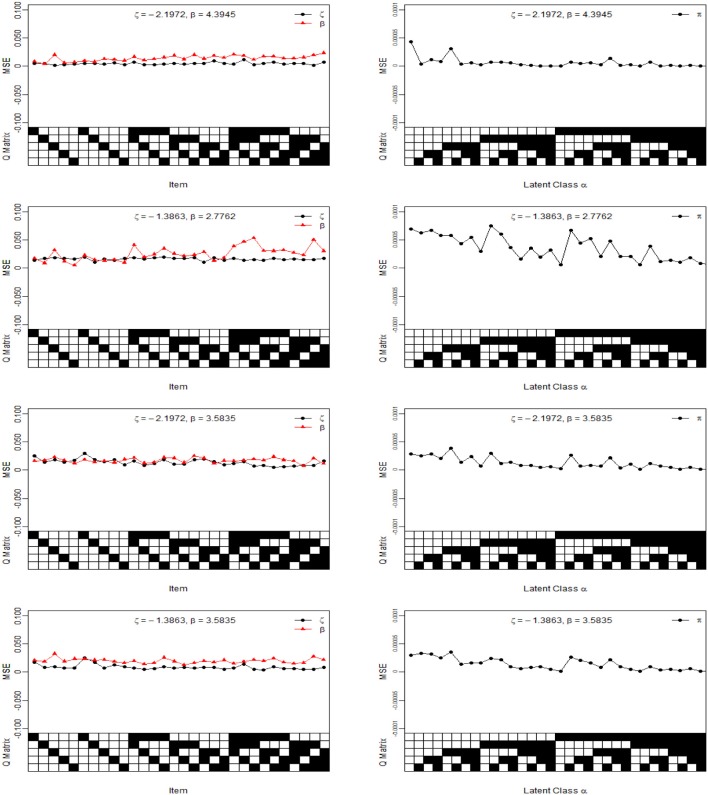
The MSE of intercept, interaction and class membership probability parameters under four different diagnosticity cases. The *Q* Matrix denotes the skills required for each item along the *x* axis, where the black square = “1” and white square = “0.” The α_*ck*_ denotes the examinee who belongs to the *c*th latent class whether has mastered *k*th skill, where the black square = “1” for the presence of a skill and white square = “0” for the absence of a skill, $\alpha_{c}={\left(\alpha_{c1},\ldots ,\alpha_{cK}\right)}^{\prime }$. Note the MSE values are estimated from 25 replications.

For illustration purposes, we only show the Bias and MSE of ***ς***, ***β***, and ***π*** for the four noise levels based on 1,000 sample sizes in Figures 2, 3. In the four noise levels, the Bias of ***ς***, ***β***, and ***π*** are near the zero values. However, the MSE of ***ς*** and ***β*** increase as the number of attributes required by the item increases. In the low noise level, the performances of the recovery for ***ς*** and ***β*** are well-based on the results of MSE, and the MSE of ***ς*** and ***β*** are <0.0250. The performances for the high noise level are worst in the four diagnosticity cases. Moreover, we find that when the item tests a attribute, the MSE of ***ς*** is not much different from that of ***β***. However, the MSE of ***β*** is greater than that of ***ς*** when the item requires multiple attributes. The reason is due to a fact that the number of examinees for η_*ij*_ = 1 is almost equal to that of η_*ij*_ = 0 when the item tests a attribute, which is accurate for estimating the ***ς*** and ***β***. Along with the increase in the attributes required by the item, the number of examinees for η_*ij*_ = 1 reduces and the number of examinees for η_*ij*_ = 0 increases, thus resulting in the MSE of ***β*** higher than that of ***ς***. Note that the MSE of ***β*** is dependent on the number of examinees for η_*ij*_ = 1.

The average Bias and MSE for ***ς***, ***β***, and ***π*** based on eight different simulation conditions are shown in Table 2. The following conclusions can be obtained. (1) Given a noise level, when the number of examinees increases from 1,000 to 2,000, the average MSE for ***ς*** and ***β*** show a decreasing trend. More specifically, when the number of examinees increases from 1,000 to 2,000, in the case of low noise level (LNL), the average MSE of ***ς*** decreases from 0.048 to 0.034, the average MSE of ***β*** decreases from 0.0141 to 0.0107. In the case of high noise level (HNL), the average MSE of ***ς*** decreases from 0.0163 to 0.0117, the average MSE of ***β*** decreases from 0.0254 to 0.0239. In the case of the slipping higher than the guessing (SHG), the average MSE of ***ς*** decreases from 0.0139 to 0.0078, the average MSE of ***β*** decreases from 0.0172 to 0.0159. In the case of the guessing higher than the slipping (GHS), the average MSE of ***ς*** decreases from 0.0088 to 0.0041, the average MSE of ***β*** decreases from 0.0198 to 0.0181. (2) Given a noise level, when the number of examinees increases from 1,000 to 2,000, In the case of four kinds of noises, the average MSE of ***π*** are basically the same and close to 0 under the conditions of four noise levels. (3) Compared with the other three noise level, the average MSE of ***ς*** and ***β*** are largest at high noise level. In summary, the Bayesian algorithm provides accurate estimates for ***ς***, ***β***, and ***π*** in term of various numbers of examinees.

**Table 2:** The average Bias and MSE for ***ς***, ***β***, and ***π***.

	**Number of examinees 1,000**			
	**LNL (b1)**	**HNL (b2)**	**SHG (b3)**	**GHS (b4)**
**BIAS**				
***ς***	0.0023	0.0046	0.0042	−0.0039
***β***	−0.1077	−0.1016	−0.0235	−0.0248
***π***	−0.0000	−0.0000	−0.0000	−0.0000
**MSE**				
***ς***	0.0048	0.0163	0.0139	0.0088
***β***	0.0141	0.0254	0.0172	0.0198
***π***	0.0000	0.0000	0.0000	0.0000
	**Number of examinees 2,000**			
**BIAS**				
***ς***	0.0089	0.0089	0.0023	−0.0020
***β***	−0.0890	0.0588	−0.0003	−0.0041
***π***	−0.0000	0.0000	−0.0000	0.0000
**MSE**				
***ς***	0.004	0.0117	0.0078	0.0041
***β***	0.0107	0.0239	0.0159	0.0181
***π***	0.0000	0.0000	0.0000	0.0000

*Note that the Bias and MSE denote the average Bias and MSE for the parameters. **ς** represents all intercept parameters, **β** represents all interaction parameters, **π** represents all class membership probabilities parameters*.

### 4.2. Simulation 2

In this simulation study, we compare MH algorithm and PGGSA from two aspects: the accuracy and convergence. We consider 1,000 examinees to answer 30 items, and the number of the attribute is set equal to *K* = 5. The true values of ζ_*j*_ and β_*j*_ are set equal to −2.1972 and 4.3945 for each item. The corresponding true values of *s*_*j*_ and *g*_*j*_ are equal to 0.1 for each item. The class membership probabilities are flat though all classes, i.e., $\pi_{c}=\frac{1}{2^{K}},$ *c* = 1, …, *C*, where *C* = 2^*K*^. We specify the following non-informative priors to the PGGSA and MH algorithm: $\zeta_{j}\sim N\left(-2.1972,10^{5}\right),\beta_{j}\sim N\left(4.3945,10^{5}\right)\text{ I }\left(\beta_{j}>0\right)$ and (π_1_, …, π_*C*_) ~ Dirichlet(1, …, 1).

It is known that an improper proposal distribution for MH algorithm can seriously reduce the acceptance probability of sampling. Most of the posterior samples are rejected. Therefore, the low sampling efficiency is usually unavoidable, and the reduction in the number of valid samples may lead to incorrect inference results. In contrast, our PGGSA takes the acceptance probability as 1 to draw the samples from fully condition posterior distributions. The following proposal distributions for the intercept and interaction parameters are considered in the process of implementing MH algorithm. The sampling details of MH algorithm, see Appendix. Note that the class membership probabilities are updated through the same way for the PGGSA and MH algorithms.

- Case 1: $\varsigma_{j}\sim N\left(\varsigma_{j}^{\left(r\right)},0.1\right),$ $\beta_{j}\sim N\left(\beta_{j}^{\left(r\right)},0.1\right)\text{ I }\left(\beta_{j}>0\right).$
- Case 2: $\varsigma_{j}\sim N\left(\varsigma_{j}^{\left(r\right)},1\right),\beta_{j}\sim N\left(\beta_{j}^{\left(r\right)},1\right)\text{ I }\left(\beta_{j}>0\right).$

**Figure 4:**
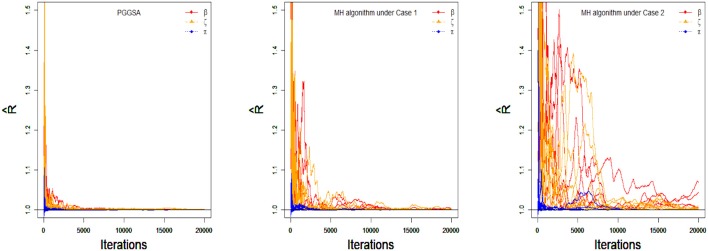
The trace plots of PSRF values for the simulation study 2.

To compare the convergence of all parameters for the PGGSA and MH algorithm with different proposal distributions, the convergence of item and class membership probability parameters are evaluated by judging whether the values of PSRF are <1.1. From Figure 4, we find that the intercept, interaction and class membership probability parameters have already converged at the 5,000 step iterations for the PGGSA. The fastest convergence is the class membership probability parameters followed by intercept parameters. For the MH algorithm, some parameters do not converge after 5,000 step iterations for the proposal distributions with the variances of 0.1. The convergence of the proposal distributions with the variances of 1 is worse than the convergence of the proposal distributions with the variances of 0.1, even some parameters do not reach convergence at the end of the 10,000 step iterations. Moreover, the Bias and MSE are used to evaluate the performances of the two algorithms in Table 3. It has been proved that the selection of the proposal distribution has an important influence on the accuracy of parameter estimation. The process of finding the proper turning parameter is time consuming. In addition, we investigate the efficiency of the two algorithms from the perspective of the time consumed by implementing them. On a desktop computer [Intel(R) Xeon(R) E5-2695 V2 CPU] with 2.4 GHz dual core processor and 192 GB of RAM memory, PGGSA and MH algorithm, respectively consume 3.6497 and 4.7456 h when Markov chain are run for 20,000 iterations for a replication experiment, where MH algorithm is used to implement the Case 1. In summary, PGGSA is more effective than MH algorithm in estimating model parameters.

**Table 3:** Evaluating accuracy of parameter estimation using the two algorithms in the simulation study 2.

	**PGGSA**		**MH algorithm under Case 1**		**MH algorithm under Case 2**	
	**Bias**	**MSE**	**Bias**	**MSE**	**Bias**	**MSE**
***ς***	0.0023	0.0048	0.0016	0.0069	0.0021	0.0081
***β***	−0.1077	0.0141	−0.1042	0.0152	−0.1087	0.0174
***π***	−0.0000	0.0000	−0.0007	0.0005	−0.0004	0.0011

*Note that the Bias and denote the average Bias and MSE for the parameters. **ς** represents all intercept parameters, **β** represents all interaction parameters, **π** represents all latent class probabilities parameters*.

### 4.3. Simulation 3

This simulation study is to show that PGGSA is sufficiently flexible to recover various prior distributions for the item and class membership probability parameters. The simulation design is as follows:

The number of the examinees is *N* = 1, 000, and the test length is *J* = 30, and the number of the attributes is set equal to *K* = 5. The true values of item intercept and interaction parameters are −2.1972 and 4.3945 for each item at low noise level. The class membership probabilities are flat though all classes, i.e., $\pi_{c}=\frac{1}{2^{K}},$ *c* = 1, …, *C*, where *C* = 2^*K*^.

The non-informative Dirichlet prior distribution is employed for the class membership probabilities ***π***. I.e., (π_1_, …, π_*C*_) ~ Dirichlet (1, …, 1), and two kinds of prior distributions are considered for the intercept and interaction parameters:

1. Informative prior: **Type I**: ζ_*j*_ ~ *N*(−2.1972, 0.5), β_*j*_ ~ *N*(4.3945, 0.5)I(β_*j*_ > 0); **Type II**: ζ_*j*_ ~ *N*(−2.1972, 1), β_*j*_ ~ *N*(4.3945, 1) I (β_*j*_ > 0);
2. Non-informative prior: **Type III**: $\zeta_{j}\sim N\left(-2.1972,10^{3}\right),$ $\beta_{j}\sim N\left(4.3945,10^{3}\right)\text{ I }\left(\beta_{j}>0\right);$ **Type IV**: $\zeta_{j}\sim N\left(-2.1972,10^{5}\right),\beta_{j}\sim N\left(4.3945,10^{5}\right)\text{ I }\left(\beta_{j}>0\right).$

PGGSA is iterated 20,000 times. The first 10,000 iterations are discarded as burn-in period. 25 replications are considered in this simulation study. The PSRF values of all parameters for each simulation condition are <1.1. The Bias and MSE of the ***ζ***, ***β***, and ***π*** based on two kinds of prior distributions are shown in Table 4.

**Table 4:** Evaluating the accuracy of parameters based on different prior distributions in the simulation study 3.

**Type of prior**	**Evaluation index**	**ς**	**β**	**π**
Type I	Bias	0.0024	−0.1044	−0.0000
	MSE	0.0047	0.0134	0.0000
Type II	Bias	0.0026	−0.1059	−0.0000
	MSE	0.0047	0.0138	0.0000
Type III	Bias	0.0022	−0.1068	−0.0000
	MSE	0.0048	0.0140	0.0000
Type IV	Bias	0.0023	−0.1077	−0.0000
	MSE	0.0048	0.0141	0.0000

*Note that the Bias and denote the average Bias and MSE for the parameters. **ς** represents all intercept parameters, **β** represents all interaction parameters, **π** represents all latent class probabilities parameters*.

#### 4.3.1. Result Analysis

From Table 4, we find that the Bias and MSE of ***ς***, ***β*** and ***π*** are almost the same under different prior distributions. More specifically, the Bias of ***ς*** ranges from 0.0022 to 0.026, ***β*** ranges from −0.1077 to −0.1044, and the Bias of ***π*** under the two kinds of prior distributions is equal to −0.0000. In addition, the MSE of ***ς*** ranges from 0.0047 to 0.0048, ***β*** ranges from 0.0134 to 0.0141, and the MSE of ***π*** under the two kinds of prior distributions is equal to −0.0000. This shows that the accuracy of parameter estimation can be guaranteed by PGGSA, no matter what the informative prior or non-informative distributions are chosen.

### 4.4. Simulation 4

The main purpose of this simulation study is to compare PGGSA and Culpepper (2015)'s Gibbs sampling algorithm (Geman and Geman, 1984; Tanner and Wong, 1987; Gelfand and Smith, 1990; Albert, 1992; Damien et al., 1999; Béguin and Glas, 2001; Sahu, 2002; Bishop, 2006; Fox, 2010; Chen et al., 2018; Lu et al., 2018) on the attribute classification accuracy and the estimation accuracy of class membership probability parameter (***π***).

The number of the examinees is *N* = 1, 000. Considering the test length is *J* = 30, and the number of the attribute is set equal to *K* = 5. The *Q-*matrix is shown in Table 1. Four noise levels are considered in this simulation, i.e., LNL, HNL, SHG, and GHS. The true values of item parameters under the four noise levels, see the simulation study 1. For the true values of the class membership probabilities, we only consider the most general case that the class membership probabilities are flat though all classes, i.e., $\pi_{c}=\frac{1}{2^{K}},$ *c* = 1, …, *C*, where *C* = 2^*K*^.

For the prior distributions of the two algorithms, we use the non-informative prior distributions to eliminate the influence of the prior distributions on the posterior inference. The non-informative Dirichlet prior distribution is employed for the class membership probabilities ***π***. I.e., (π_1_, …, π_*C*_) ~ Dirichlet (1, …, 1), and the non-informative prior distributions of item parameters under the two algorithms based on the four noise levels are set as follows

- (**LNL case**): PGGSA: $\zeta_{j}\sim N\left(-2.1972,10^{5}\right),\beta_{j}\sim N\left(4.3945,10^{5}\right)\text{ I }\left(\beta_{j}>0\right).$ v.s. Gibbs algorithm: *s*_*j*_ ~ *Beta*(1, 1), *g*_*j*_ ~ *Beta*(1, 1) I (*g*_*j*_ < 1 − *s*_*j*_);
- (**HNL case**): PGGSA: $\zeta_{j}\sim N\left(-1.3863,10^{5}\right),\beta_{j}\sim N\left(2.7726,10^{5}\right)\text{ I }\left(\beta_{j}>0\right).$ v.s. Gibbs algorithm: *s*_*j*_ ~ *Beta*(1, 1), *g*_*j*_ ~ *Beta*(1, 1) I (*g*_*j*_ < 1 − *s*_*j*_);
- (**SHG case**): PGGSA: $\zeta_{j}\sim N\left(-2.1972,10^{5}\right),\beta_{j}\sim N\left(3.5835,10^{5}\right)\text{ I }\left(\beta_{j}>0\right).$ v.s. Gibbs algorithm: *s*_*j*_ ~ *Beta*(1, 1), *g*_*j*_ ~ *Beta*(1, 1) I (*g*_*j*_ < 1 − *s*_*j*_);
- (**GHS case**): PGGSA: $\zeta_{j}\sim N\left(-1.3863,10^{5}\right),\beta_{j}\sim N\left(3.5835,10^{5}\right)\text{ I }\left(\beta_{j}>0\right).$ v.s. Gibbs algorithm: *s*_*j*_ ~ *Beta*(1, 1), *g*_*j*_ ~ *Beta*(1, 1) I (*g*_*j*_ < 1 − *s*_*j*_).

PGGSA and Gibbs algorithm are iterated 20,000 times. The first 10,000 iterations are discarded as burn-in period for the two algorithms. Twenty-five replications are considered for the two algorithms in this simulation study. The PSRF values of all parameters for each simulation condition are <1.1. Culpepper's the R “dina” package is used to implement the Gibbs sampling.

The correct pattern classification rate (CPCR), the average attribute match rate (AAMR) are used as the evaluation criteria to evaluate the attributes. These statistics are defined as

(26) $$\begin{array}{l} \text{CPCR}=\frac{1}{N}\overset{N}{\underset{i=1}{\sum }}\text{ I }\left(\alpha_{i}=\hat{\alpha_{i}}\right),\text{ AAMA}=\frac{1}{N\times K}\overset{N}{\underset{i=1}{\sum }}\overset{K}{\underset{k=1}{\sum }}\text{ I }\left(\alpha_{ik}=\hat{\alpha_{ik}}\right). \end{array}$$

where $\hat{\alpha_{i}}={\left(\hat{\alpha_{i1}},\alpha_{i2},\ldots ,\alpha_{iK}\right)}^{\prime }$ represents examinee *i*′s estimated attribute patterns. Next, the evaluation results of the accuracy of the two algorithms for attribute patterns and class membership probability parameters are shown in Table 5.

**Table 5:** Evaluating accuracy of attribute and class membership probability parameter estimations using PGGSA and Gibbs algorithm in the simulation study 4.

		**Attribute(α)**		**CMP(π)**	
**Noise level**	**Algorithm**	**CPCR**	**AAMA**	**Bias**	**MSE**
LNL	PGGSA	0.8740	0.9693	−0.0000	0.0000
	Gibbs	0.8722	0.9688	−0.0000	0.0000
HNL	PGGSA	0.5643	0.8696	−0.0000	0.0000
	Gibbs	0.5697	0.8718	−0.0000	0.0000
SHG	PGGSA	0.7480	0.9336	−0.0000	0.0000
	Gibbs	0.7429	0.9308	−0.0000	0.0000
GHS	PGGSA	0.8436	0.9310	−0.0000	0.0000
	Gibbs	0.8484	0.9338	−0.0000	0.0000

*Note that the CMP denotes the class membership probability. Bias and MSE denote the average Bias and MSE for the class membership probability parameters*.

In Table 5, we find that the results of the attributes classification accuracy (CPCR and AAMA criteria) are basically the same for PGGSA and Gibbs algorithm under four kinds of noise levels. More specifically, the values of CPCR and AAMA for two algorithms under the HNL case are lowest. At the LNL case, the values of CPCR and AAMA for two algorithms are the highest. In addition, the CPCR value for the SHG case is lower than the CPCR value for the GHS, while the corresponding AAMA values are basically the same for the SHG case and GHS case. This indicates that slipping parameters (***s***) have important influence on the CPCR. In term of the two algorithms, the Bias and MSE of the classification membership parameters (***π***) are basically the same and close to zero under the four noise levels.

## 5. Empirical Example

In this example, a fraction subtraction test data is analyzed based on Tatsuoka (1990), Tatsuoka (2002), and de la Torre and Douglas (2004). The middle school students of 2,144 take part in this test to response 15 fraction subtraction items, where five attributes are measured, including subtract basic fractions, reduce and simplify, separate whole from fraction, borrow from whole, and convert whole to fraction. We choose 536 of 2,144 students in this study. These students are divided into 2^5^ latent classes based on the five attributes. The reparameterized DINA model is used to analyze the cognitive response data.

**Figure 5:**
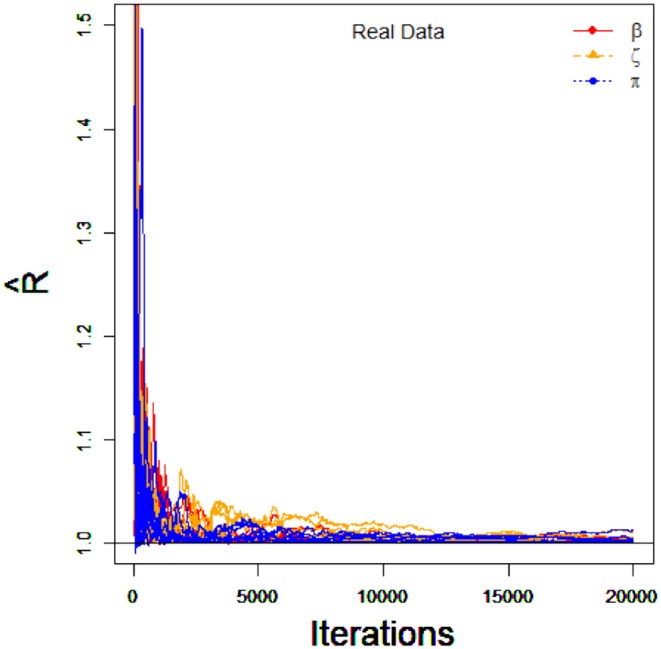
The trace plots of PSRF values for the real data.

The priors of parameters are also the same as the simulation 1. I.e., the non-informative priors are used in this empirical example analysis. To implement PGGSA, chains of length 20,000 with an initial burn-in period 10,000 are chosen. The PSRF is used to evaluate the convergence of each parameters. The trace plots of PSRF values for all parameters is shown in Figure 5. We find that the values of PSRF are <1.1.

The *Q* matrix, the expected *a posteriori* (EAP) estimators of the item parameters, the corresponding standard deviation (SD), and 95% highest posterior density intervals (HPDIs) of these item parameters are shown in Table 6. Based on the Table 6, we transform intercept and interaction parameters into traditional slipping and guessing parameters to analyze item characteristics. We find that the expected *a posteriori* (EAP) estimations of the five items with the lowest slipping are item 3, item 8, item 9, item 10, and item 11 in turn. The EAP estimations of slipping parameters for the five items are 0.0461, 0.0585, 0.0708, 0.0754, and 0.1039. This shows that these items are not easy to slipping compared with the other ten items. In addition, the EAP estimations of five items with the highest guessing are item 3, item 2, item 8, item 10, and item 11 in turn. The EAP estimations of guessing parameters for the five items are 0.2271, 0.2143, 0.2069, 0.1790, and 0.1441. Furthermore, we find that items 3, 8, 10, and 11 have low slipping parameters and high guessing parameters, which indicates that these items are more likely to be guessed correctly.

**Table 6:** The *Q* matrix design and MCMC estimations of ***ς*** and ***β***.

	**Attribute(***Q***Matrix)**					$\hat{\varsigma }$			$\hat{\beta }$		
**Item**	**α_1_**	**α_2_**	**α_3_**	**α_4_**	**α_5_**	**EAP**	***SD***	**HPDI**	**EAP**	***SD***	**HPDI**
1	1	0	0	0	0	−2.3274	0.0277	[−2.4998, −1.9766]	3.3884	0.0662	[2.8484, 3.8721]
2	1	1	1	1	0	−1.2990	0.0225	[−1.5639, −1.0087]	3.4200	0.0947	[2.8714, 4.0615]
3	1	0	0	0	0	−1.2247	0.0276	[−1.5357, −1.0000]	4.2999	0.0294	[3.9575, 4.4999]
4	1	1	1	1	1	−1.8944	0.0358	[−2.2841, −1.5472]	3.8815	0.1217	[3.2857, 4.4977]
5	0	0	1	0	0	−1.7971	0.1042	[−2.4667, −1.2948]	2.9899	0.1145	[2.5007, 3.6131]
6	1	1	1	1	0	−2.3961	0.0113	[−2.4999, −2.1653]	3.7058	0.0817	[3.1377, 4.2461]
7	1	1	1	1	0	−2.1109	0.0322	[−2.4999, −1.8117]	4.3549	0.0223	[4.0401, 4.4998]
8	1	1	0	0	0	−1.3433	0.0409	[−1.7158, −1.0005]	4.1817	0.0558	[3.7427, 4.4999]
9	1	0	1	0	0	−1.6266	0.0566	[−2.0725, −1.1512]	4.2735	0.0384	[3.8794, 4.4998]
10	1	0	1	1	1	−1.5226	0.0246	[−1.8180, −1.2110]	4.1072	0.0796	[3.5678, 4.4999]
11	1	0	1	0	0	−1.7813	0.0681	[−2.3048, −1.2903]	4.0454	0.0884	[3.5121, 4.4999]
12	1	0	1	1	0	−2.3802	0.0119	[−2.4998, −2.1534]	4.2212	0.0481	[3.7945, 4.4994]
13	1	1	1	1	0	−1.8221	0.0399	[−2.2142, −1.4328]	3.5878	0.1009	[2.9818, 4.1937]
14	1	1	1	1	1	−2.4279	0.0058	[−2.4999, −2.2647]	3.8646	0.0982	[3.3310, 4.4741]
15	1	1	1	1	0	−2.4298	0.0060	[−2.4999, −2.2551]	4.0033	0.0765	[3.5339, 4.4946]

*Note that α_1_ denotes the skill of subtract basic fractions, α_2_ denotes the skill of reduce and simplify, α_3_ denotes the skill of separate whole from fraction, α_4_ denotes the skill of borrow from whole, α_5_ denotes the skill of convert whole to fraction. EAP denotes expected a posteriori estimator. SD denotes standard deviation. HPDI denotes 95% highest posterior density intervals (HPDI)*.

The EAP estimations of the class membership probabilities, ${\hat{\pi }}_{c}$, *c* = 1, …, 32, and the corresponding SD and 95% HPDI are reported in Table 7. The top five classes that the majority of examinees are classified into these classes are respectively “11111,”“11101,”“11100,”“11110,” and “00010.”The estimation results show that ${\hat{\pi }}_{32}=$ 34.142% of the examinees have mastered all the five skills, and ${\hat{\pi }}_{28}=$ 11.119% of the examinees have mastered the four skills except the skill of borrow from whole, and the examinees who only have mastered the three skills of subtract basic fractions, reduce and simplify, separate whole from fraction account for ${\hat{\pi }}_{17}=$ 10.146%, and ${\hat{\pi }}_{27}=$ 9.680% of the examinees have mastered the four skills except the skill of convert whole to fraction, and the examinees who only have mastered a skill of skill of borrow from whole account for ${\hat{\pi }}_{3}=$ 2.001%. In addition, among the thirty-two classes, the class with the lowest number of the examinees is ${\hat{\pi }}_{30}=$ 0.429%. I.e., when the examinees have mastered the skills of subtract basic fractions, separate whole from fraction, borrow from whole, and convert whole to fraction, the proportion of examinees who do not master the skill of reduce and simplify is very low. According to the ${\hat{\pi }}_{3}=$ 1.743% and ${\hat{\pi }}_{30}=$ 0.429%, we find that the skill of reduce and simplify is easier to master than the other four skills.

**Table 7:** The posterior probability distribution of the latent class parameters for the Fraction Subtraction Test.

**Latent classes**					$\hat{\pi }$		
**α_1_**	**α_2_**	**α_3_**	**α_4_**	**α_5_**	**EAP**	***SD***	**HPDI**
0	0	0	0	0	1.909%	0.0003	[0.0000, 0.0542]
1	0	0	0	0	0.766%	0.0000	[0.0000, 0.0208]
0	1	0	0	0	1.743%	0.0002	[0.0000, 0.0504]
0	0	1	0	0	1.299%	0.0001	[0.0000, 0.0367]
0	0	0	1	0	2.001%	0.0002	[0.0000, 0.0533]
0	0	0	0	1	1.790%	0.0002	[0.0000, 0.0540]
1	1	0	0	0	0.677%	0.0000	[0.0000, 0.0190]
1	0	1	0	0	1.898%	0.0001	[0.0000, 0.0443]
1	0	0	1	0	0.756%	0.0000	[0.0000, 0.0203]
1	0	0	0	1	0.822%	0.0000	[0.0000, 0.0222]
0	1	1	0	0	1.162%	0.0001	[0.0000, 0.0339]
0	1	0	1	0	1.808%	0.0002	[0.0000, 0.0507]
0	1	0	0	1	1.943%	0.0003	[0.0000, 0.0567]
0	0	1	1	0	1.242%	0.0001	[0.0000, 0.0330]
0	0	1	0	1	1.165%	0.0001	[0.0000, 0.0328]
0	0	0	1	1	1.778%	0.0002	[0.0000, 0.0486]
1	1	1	0	0	10.146%	0.0039	[0.0002, 0.2029]
1	1	0	1	0	0.709%	0.0000	[0.0000, 0.0198]
1	1	0	0	1	0.764%	0.0000	[0.0000, 0.0205]
1	0	1	1	0	0.546%	0.0000	[0.0000, 0.0140]
1	0	1	0	1	1.782%	0.0001	[0.0000, 0.0419]
1	0	0	1	1	0.751%	0.0000	[0.0000, 0.0201]
0	1	1	1	0	1.326%	0.0001	[0.0000, 0.0370]
0	1	1	0	1	1.181%	0.0001	[0.0000, 0.0357]
0	1	0	1	1	1.675%	0.0002	[0.0000, 0.0473]
0	0	1	1	1	1.167%	0.0001	[0.0000, 0.0335]
1	1	1	1	0	9.680%	0.0002	[0.0667, 0.1264]
1	1	1	0	1	11.119%	0.0038	[0.0001, 0.2078]
1	1	0	1	1	0.688%	0.0000	[0.0000, 0.0195]
1	0	1	1	1	0.429%	0.0000	[0.0000, 0.0119]
0	1	1	1	1	1.119%	0.0001	[0.0000, 0.0320]
1	1	1	1	1	34.142%	0.0004	[0.2998, 0.3844]

*Note that α_1_ denotes the skill of subtract basic fractions, α_2_ denotes the skill of reduce and simplify, α_3_ denotes the skill of separate whole from fraction, α_4_ denotes the skill of borrow from whole, α_5_ denotes the skill of convert whole to fraction*.

## 6. Conclusion

In this paper, a novel and effective PGGSA based on auxiliary variables is proposed to estimate the widely applied DINA model. PGGSA overcomes the disadvantages of MH algorithm, which requires to repeatedly adjust the specification of tuning parameters to achieve a certain acceptance probability and thus increases the computational burden. However, the computational burden of the PGGSA becomes intensive especially as the CDMs become more complex, when a large number of examinees or the items is considered, or a large number of the MCMC sample size is used. Therefore, it is desirable to develop a standing-alone R package associated with C++ or Fortran software for more extensive CDMs and large-scale cognitive assessment tests.

In addition, Pólya-Gamma Gibbs sampling algorithm can be used to estimate many cognitive diagnosis models, which is not limited to the DINA model. These cognitive diagnostic models include DINO (Templin and Henson, 2006), Compensatory RUM (Hartz, 2002; Henson et al., 2009), and log-linear CDM (LCDM; von Davier, 2005; Henson et al., 2009) and so on. More specifically, first of all, the parameters of these cognitive diagnosis models are reparameterized, and then the logit link function is used to link these parameters with the response. Further, we can use Pólya-Gamma Gibbs sampling algorithm to estimate these reparameterized cognitive diagnosis models. Discussions of the reparameterized cognitive diagnosis models based on logit link function, see Henson et al. (2009).

## Data Availability Statement

Publicly available datasets were analyzed in this study. This data can be found here: https://cran.r-project.org/web/packages/CDM/index.html.

## Author Contributions

JZ completed the writing of the article. ZZ and JZ provided article revisions. JZ, JL, and ZZ provided the key technical support. JT provided the original thoughts.

### Conflict of Interest

The authors declare that the research was conducted in the absence of any commercial or financial relationships that could be construed as a potential conflict of interest.
