# Supplementary material for: Bayesian Estimation of the DINA Model With Pólya-Gamma Gibbs Sampling
Source: Front Psychol. 2020 Mar 10;11:384. doi: 10.3389/fpsyg.2020.00384 (PMC7076190; doi:10.3389/fpsyg.2020.00384)
Supplement: Supplementary file 1 [file Data_Sheet_1.pdf]

Appendix

Sampling processes of Metropolis-Hastings algorithm.

Step 1: Sampling the intercept parameter ς_j for each item j. The prior distribution of ς_j is assumed to follow the normal distribution $\varsigma_j \sim N\left(\mu_\varsigma, \sigma_\varsigma^2\right)$. The new value ς_j^* which is sampled from a normal distribution $N\left(\varsigma_j^{(r-1)}, s_{\varsigma_j}^2\right)$. Therefore, the probability of acceptance $\alpha\left(\varsigma_j^{(r-1)}, \varsigma_j^*\right)$ can be written as

$$\min\left\{1, \frac{\prod_{i=1}^{N} p\left(Y_{ij} = y_{ij} \middle| \varsigma_j^*, \beta_j^{(r-1)}, \boldsymbol{\alpha}_i^{(r-1)} = \boldsymbol{\alpha}_c^{(r-1)}\right) p_{prior}\left(\varsigma_j^*\right) p_{proposal}\left(\varsigma_j^{(r-1)} \middle| \varsigma_j^*, s_{\varsigma_j}^2\right)}{\prod_{i=1}^{N} p\left(Y_{ij} = y_{ij} \middle| \varsigma_j^{(r-1)}, \beta_j^{(r-1)}, \boldsymbol{\alpha}_i^{(r-1)} = \boldsymbol{\alpha}_c^{(r-1)}\right) p_{prior}\left(\varsigma_j^{(r-1)}\right) p_{proposal}\left(\varsigma_j^* \middle| \varsigma_j^{(r-1)}, s_{\varsigma_j}^2\right)}\right\}, \tag{1}$$

otherwise, $\varsigma_j^{(r-1)} = \varsigma_j^*$.

Step 2: Sampling the interaction parameter β_j for each item j. The prior distribution of β_j is assumed to follow the truncated normal distribution $\beta_j \sim N\left(\mu_\beta, \sigma_\beta^2\right) \mathrm{I}\left(\beta_j > 0\right)$. The new value β_j^* which is sampled from a normal distribution $N\left(\beta_j^{(r-1)}, s_{\beta_j}^2\right)$. Therefore, the probability of acceptance $\alpha\left(\beta_j^{(r-1)}, \beta_j^*\right)$ can be written as

$$\min\left\{1, \frac{\prod_{i=1}^{N} p\left(Y_{ij} = y_{ij} \middle| \varsigma_j^{(r)}, \beta_j^*, \boldsymbol{\alpha}_i^{(r-1)} = \boldsymbol{\alpha}_c^{(r-1)}\right) p_{prior}\left(\beta_j^*\right) p_{proposal}\left(\beta_j^{(r-1)} \middle| \beta_j^*, s_{\beta_j}^2\right)}{\prod_{i=1}^{N} p\left(Y_{ij} = y_{ij} \middle| \varsigma_j^{(r)}, \beta_j^{(r-1)}, \boldsymbol{\alpha}_i^{(r-1)} = \boldsymbol{\alpha}_c^{(r-1)}\right) p_{prior}\left(\beta_j^{(r-1)}\right) p_{proposal}\left(\beta_j^* \middle| \beta_j^{(r-1)}, s_{\beta_j}^2\right)}\right\}, \tag{2}$$

otherwise, $\beta_j^{(r-1)} = \beta_j^*$.

Step 3: Sampling the interaction parameter β_j for each item j. The prior distribution of β_j is assumed to follow the truncated normal distribution $\beta_j \sim N\left(\mu_\beta, \sigma_\beta^2\right) \mathrm{I}\left(\beta_j > 0\right)$. The new value β_j^* which is sampled from a normal distribution $N\left(\beta_j^{(r-1)}, s_{\beta_j}^2\right)$. Therefore,

the probability of acceptance $\alpha\left(\beta_j^{(r-1)}, \beta_j^*\right)$ can be written as

$$\min\left\{1, \frac{\prod_{i=1}^{N} p\left(Y_{ij} = y_{ij} \left| \varsigma_j^{(r)}, \beta_j^*, \boldsymbol{\alpha}_i^{(r-1)} = \boldsymbol{\alpha}_c^{(r-1)}\right.\right) p_{prior}\left(\beta_j^*\right) p_{proposal}\left(\beta_j^{(r-1)} \left| \beta_j^*, s_{\beta_j}^2\right.\right)}{\prod_{i=1}^{N} p\left(Y_{ij} = y_{ij} \left| \varsigma_j^{(r)}, \beta_j^{(r-1)}, \boldsymbol{\alpha}_i^{(r-1)} = \boldsymbol{\alpha}_c^{(r-1)}\right.\right) p_{prior}\left(\beta_j^{(r-1)}\right) p_{proposal}\left(\beta_j^* \left| \beta_j^{(r-1)}, s_{\beta_j}^2\right.\right)}\right\}, \tag{3}$$

otherwise, $\beta_j^{(r-1)} = \beta_j^*$.

Step 4: Sampling the attribute vector $\boldsymbol{\alpha}_i$ for each examinee i. Given $\boldsymbol{Y}$, $\boldsymbol{W}$, $\boldsymbol{\varsigma}$ and $\boldsymbol{\beta}$, we can draw the ith examinee's attribute vector $\boldsymbol{\alpha}_i$ from the following multinomial distribution

$$\boldsymbol{\alpha}_i \left| \boldsymbol{Y}_i, \boldsymbol{W}_i, \boldsymbol{\varsigma}, \ \boldsymbol{\beta} \right. \sim \text{Multinomial}\left(1, \left[\lambda_{i1}, ..., \lambda_{iC}\right]\right).$$

where the probability of the attribute vector $\boldsymbol{\alpha}_i$ belonging to the category $c\,(c = 1, ..., C)$ can be written as

$$\lambda_{ic} = P\left(\boldsymbol{\alpha}_i = \boldsymbol{\alpha}_c \left| \boldsymbol{Y}_i, \boldsymbol{W}_i, \boldsymbol{\varsigma}, \boldsymbol{\beta}, \boldsymbol{\pi}\right.\right) = \frac{\pi_c P\left(\boldsymbol{Y}_i \left| \boldsymbol{\alpha}_i = \boldsymbol{\alpha}_c, \boldsymbol{\varsigma}, \boldsymbol{\beta}\right.\right) f\left(\boldsymbol{W}_i \left| \boldsymbol{\alpha}_i = \boldsymbol{\alpha}_c, \boldsymbol{\varsigma}, \boldsymbol{\beta}\right.\right)}{\sum_{c=1}^{C} \pi_c P\left(\boldsymbol{Y}_i \left| \boldsymbol{\alpha}_i = \boldsymbol{\alpha}_c, \boldsymbol{\varsigma}, \boldsymbol{\beta}\right.\right) f\left(\boldsymbol{W}_i \left| \boldsymbol{\alpha}_i = \boldsymbol{\alpha}_c, \boldsymbol{\varsigma}, \boldsymbol{\beta}\right.\right)} \tag{4}$$

Step 5: Sampling the latent class probabilities $\boldsymbol{\pi}$, The prior of $\boldsymbol{\pi}$ is assumed to follow the Dirichlet distribution. That is, $\boldsymbol{\pi} = (\pi_1, ..., \pi_C) \sim \text{Dirichlet}(\delta_0, ..., \delta_0)$. The full condition distribution of the latent class probabilities $\boldsymbol{\pi}$ can be written as

$$\boldsymbol{\pi} \left| \boldsymbol{\alpha}_1, ..., \boldsymbol{\alpha}_C \right. \sim \text{Dirichlet}\left(\delta_0 + \sum_{i=1}^{N} I\left(\boldsymbol{\alpha}_i = \boldsymbol{\alpha}_1\right), ..., \delta_0 + \sum_{i=1}^{N} I\left(\boldsymbol{\alpha}_i = \boldsymbol{\alpha}_C\right)\right). \tag{5}$$
